# Local ketorolac infiltration for postoperative pain in open trigger finger surgery: a randomized controlled trial

**DOI:** 10.1186/s12891-024-07856-6

**Published:** 2024-09-17

**Authors:** Thanat Auwattanamongkol, Panai Laohaprasitiporn, Yuwarat Monteerarat, Roongsak Limthongthang, Torpon Vathana

**Affiliations:** Department of Orthopaedic Surgery, Faculty of Medicine Siriraj Hospital, Mahidol University, Bangkok, Thailand

**Keywords:** Trigger finger, Trigger digit, Local NSAIDs infiltration, Open trigger finger surgery, Postoperative pain

## Abstract

**Background:**

Multimodal analgesia is crucial for effective postoperative pain management in minor hand surgeries, enhancing patient satisfaction. The use of local wound infiltration with Ketorolac as an adjuvant pain management strategy is proposed for open trigger finger release surgery. This study aims to compare pain scores and functional outcomes between local wound infiltration with Ketorolac and oral non-steroidal anti-inflammatory drugs.

**Methods:**

This study is a double-blind, parallel design, randomized controlled trials. Sixty-nine patients underwent trigger finger surgery between December 2021 and October 2022 were randomized into one of three groups: oral Ibuprofen alone group, local Ketorolac alone group and local Ketorolac with oral Ibuprofen group. The assessment included postoperative numeric rating scale (NRS) pain score, Disabilities of the Arm, Shoulder, and Hand (DASH) score, grip strength, mobility of proximal interphalangeal (PIP) joint. and complications.

**Results:**

NRS pain scores during movement of the operated fingers were significantly lower at 6 h in local Ketorolac alone group and local Ketorolac with oral Ibuprofen group compared to oral Ibuprofen alone group. However, there were no significant differences between the groups in postoperative DASH scores, grip strength, mobility of PIP joints, and complications.

**Conclusions:**

Local infiltration of Ketorolac as an adjunct in postoperative pain management has been shown to provide superior analgesia during finger movement within the initial 6 h following trigger finger surgery, in comparison to oral NSAIDs.

**Clinical trial registration:**

Thaiclinicaltrials.org identifier: TCTR20210825002. Registered 25/08/2021. https://www.thaiclinicaltrials.org/show/TCTR20210825002

## Introduction

Trigger finger is a prevalent condition affecting the upper extremities in adult patients. Its occurrence is quite common, with an annual incidence rate of 28 cases per 100,000 individuals in the adult population [1–3]. Typically, patients suffering from trigger finger experience pain or a clicking sensation near the metacarpal head level of the hand. These symptoms can lead to difficulties in holding objects or grasping items effectively [1–3].

One of the treatment options available for patients with trigger finger is surgical intervention, which includes open release and percutaneous release of the A1 pulley. The success rate for open release of the A1 pulley ranges from 90 to 100% [2, 4, 5]. However, it is important to note that a small percentage (approximately 5–12%) of patients who undergo open surgical treatment may experience postoperative complications or prolonged symptoms [2, 5, 6]. These symptoms may include snapping, locking, or pain even after the surgery has been performed. Approximately 19% of patients may continue to experience these symptoms for more than 8 weeks after the surgical procedure [6]. The persistence of prolonged postoperative symptoms can lead to patient dissatisfaction and may also hinder the ability to perform daily activities, impacting the patient’s overall quality of life. Therefore, careful consideration and monitoring of patients after surgical treatment for trigger finger are crucial to address any potential complications and ensure optimal outcomes.

Multimodal analgesic drugs play a pivotal role in managing postoperative pain, even in minor hand surgeries [6]. Among the commonly prescribed medications for postoperative pain control in open trigger finger release, paracetamol and non-steroidal anti-inflammatory drugs (NSAIDs) are frequently used. In contrast, opioids are rarely employed for pain management in minor surgical procedures. To provide local pain control after surgery, some surgeons have adopted the approach of administering analgesic drugs through local infiltration at the surgical site. This localized method of pain management can help alleviate discomfort and enhance patient recovery following the procedure [7–11]. 

Ketorolac is an intravenous NSAID commonly prescribed for short-term postoperative pain control. Its unique properties make it nonirritating to human tissue, and its pKa of 3.5 enables it to concentrate in injured and inflamed tissues [12]. Due to these characteristics, ketorolac can be effectively used as a local infiltration at the surgical wound site, thereby enhancing postoperative pain control. Studies have shown that local infiltration of 30–60 mg of ketorolac has proven effective for providing local analgesia with minimal systemic absorption [8]. Moreover, research has indicated that ketorolac can effectively reduce early postoperative pain in various surgical procedures, including hindfoot arthrodesis, primary breast augmentation, and total knee arthroplasty [7, 9–11]. Notably, using ketorolac as a local infiltration drug has demonstrated safety, with no evidence of serious complications reported in studies [9, 10, 13, 14]. This makes ketorolac a viable option for enhancing postoperative pain control without undue risk of adverse effects.

The current evidence regarding local ketorolac infiltration for postoperative pain control in hand surgery is limited. Therefore, the objective of this randomized controlled study is to assess the effectiveness of local ketorolac infiltration as compared to conventional oral NSAIDs in managing postoperative pain in patients who have undergone open trigger finger surgery.

## Methods

### Study design

The study protocol underwent a thorough review and received approval from the Institutional Review Board of research involving human subjects. This study was performed in accordance with the Declaration of Helsinki and reported according to Consolidated Standards of Reporting Trials (CONSORT). Additionally, this research protocol was registered in the Clinical Trials Registry database to ensure transparency and adherence to ethical standards. Before enrolling in the study, all eligible participants were provided with comprehensive information about the study, its objectives, potential risks, and benefits. Informed consent was obtained from each participant, indicating their voluntary decision to participate in the research after fully understanding its implications.

This study was a parallel design, randomized controlled trial and conducted at a tertiary care hospital from December 2021 to October 2022. The study focused on patients who underwent open trigger finger release on a single digit, with the procedure performed under local anesthesia. We included patients aged between 18 and 75 years old and presenting with a Quinell grade 2–4 single trigger digit. Patients who met any of the exclusion criteria were not included in the study. The exclusion criteria encompassed patients who had undergone prior trigger finger release in the same digit, had recurrent trigger finger, had congenital trigger finger, had experienced a fracture or tendon injury on the target digit, had inflammatory joint diseases (e.g., rheumatoid arthritis or gouty arthritis), had concomitant ipsilateral carpal tunnel syndrome and/or de Quervain’s disease, had a known history of hypersensitivity to NSAIDs, had concomitant diseases that contraindicated the use of NSAIDs (e.g., chronic kidney disease stage 3, gastric ulcer, gastroesophageal reflux disease, history of gastrointestinal bleeding, coronary artery disease, cerebrovascular disease, etc.), or were pregnant or breastfeeding women.

The participants in this study were randomly assigned to one of three treatment groups using a computerized randomization process in blocks of six. The results of the randomization were placed in opaque envelopes and remained sealed until the patient was positioned on the operating table. The trial drugs were also packaged within these sealed envelopes.

Patients in the Ibuprofen alone group received a local injection of 1 ml of normal saline solution (NSS) before the surgical wound closure, and they were prescribed 400 mg of Ibuprofen in the form of 2 capsules, to be taken three times per day for 5 days. Patients in the Ketorolac alone group were locally injected with 1 ml of Ketorolac (30 mg/ml) before surgical wound closure, and for postoperative pain management, they received oral placebo in the form of 2 capsules, to be taken three times per day for 5 days. Patients in the Ketorolac with Ibuprofen group were treated with a 1 ml injection of Ketorolac (30 mg/ml) before the surgical wound closure, and they were prescribed 400 mg of Ibuprofen in the form of 2 capsules, to be taken three times per day for 5 days. All patients took the oral medications immediately after the operation.

In this clinical trial, a 1 ml injection of Ketorolac was used as one of the treatment drugs. This Ketorolac injection was odorless and colorless, and its appearance was indistinguishable from normal saline. To ensure blinding in the study, the Ibuprofen tablets for postoperative pain control were encapsulated in white capsules, making them visually indistinguishable from other drugs. Participants in the Ketorolac alone group received a placebo drug, which was a white capsule containing medical starch. Both the Ibuprofen capsule and the placebo capsule were made to look identical, further supporting the blinding process.

This clinical trial was conducted as a double-blinded study. The patients participating in the study were not informed about which drugs they received during the trial. Additionally, the surgeon who performed the trigger finger release procedures remained unaware of the specific drug assigned to each patient, either for injection before the operation or for postoperative medications. To eliminate potential biases, a trained research coordinator was responsible for carrying out all assessments and data collection.

The primary outcome of this study was the pain score, assessed using the numeric rating scale (NRS) at 48 h after the trigger finger surgery. Secondary outcomes included the NRS pain score, the Disabilities of the Arm, Shoulder, and Hand (DASH) score, grip strength, range of motion (ROM) of the proximal interphalangeal (PIP) joint of the affected finger and complications resulting from the treatment interventions. These secondary outcomes were recorded at 1 week, 2 weeks, 6 weeks and 3 months post-surgery. Additionally, NRS pain scores at 6 h and 24 h post-surgery were also documented.

### Study protocol and surgical technique

Before the operation, participants underwent a comprehensive assessment that included interviews and preoperative examinations to collect various types of data which included preoperative NRS pain score, ROM of PIP joint of the affected finger, grip strength, and DASH score.

In the operating room, the patients were positioned on the operating table with the operating arm extended. The surgical site was prepared and draped with a surgical towel to maintain a sterile environment. The patient’s view of the surgical field was obstructed by the surgical draping to maintain blinding during the procedure. A tourniquet was applied to the proximal forearm to control blood flow during the surgery. The surgeon then administered 4 ml of 1% lidocaine without adrenaline for local anesthesia at the surgical site. Once the anesthesia was effective, a 1-cm longitudinal skin incision was made, followed by a longitudinal complete release of the A1 pulley. Before closing the skin, the assistant nurse opened the sealed envelope containing the specific drug assigned to the participant’s treatment group as per randomization. This process was concealed from the surgeon to maintain blinding. The assistant nurse prepared the injection drug, using a 27-Gauge needle and 3 ml syringe. Then, 1 ml of Ketorolac or 1 ml of NSS was injected into subcutaneous tissue of the surgical site. The tourniquet was then removed. After checking for any bleeding and suturing the skin with Nylon 4 − 0, the surgical wound was covered with sterile dressing.

In all treatment groups, patients were prescribed with a standard medication regimen to manage their postoperative care. This included 500 mg of paracetamol, which could be taken as needed every 6 h to alleviate pain and discomfort. All participants were given 20 mg of omeprazole once a day for 5 days. Along with the standard medications mentioned above, patients in Ibuprofen group and Ketorolac with Ibuprofen group were administered 400 mg of Ibuprofen in the form of 2 capsules, to be taken three times per day for 5 days. For Ketorolac alone group, placebo capsules were prescribed and to be taken three times per day for 5 days.

During the postoperative period, pain scores were assessed at specific time points. At 6 h, 24 h, and 48 h after the surgery, participants were contacted by phone and asked to provide pain scores at the surgical site using the numeric rating scale (NRS), rating from 0 (no pain) to 10 (the worst pain imaginable). Participants’ compliance with the prescribed medications was also recorded. This involved tracking the total number of Ibuprofen or placebo capsules and paracetamol tablets that the participants had taken up to each specific time point. Furthermore, any complications experienced by the participants were carefully monitored and documented. These complications included local bruising, hematoma, wound dehiscence, finger numbness, and surgical site infection.

After the trigger finger surgery, participants were scheduled to visit for postoperative checkups. These visits occurred at 1 week, 6 weeks, and 3 months after the operation. During the first week visit, the surgical wound was assessed, and the stitches were completely removed. Participants’ pain score, ROM of PIP joint using digital goniometer, grip strength using Jamar handgrip dynamometer, DASH score, and any complications experienced were collected and recorded at each visit. Additionally, participants were contacted by phone 2 weeks after the operation to inquire about their pain score.

### Sample size calculation

The effect size of this study was determined from mean and standard deviation from each group. Sample size calculation was done using repeated measures analysis of variance (ANOVA) test to detect difference between treatment groups. Due to the absence of previous studies on the use of local NSAID infiltration in open trigger finger release surgery, the effect size for this study was derived from our pilot study involving 12 patients. Fifty-seven participants were required to detect the effect size of 0.376 with 90% statistical power and a significance level of 0.05. A 20% drop-out rate was expected in this study; therefore, a total of 69 participants were required for this study and randomly selected into 3 groups.

### Statistical analysis

Continuous variables were presented as mean and standard deviation when the data followed a normal distribution, or median and interquartile range when the data exhibited a nonnormal distribution. The Kruskal-Wallis ANOVA test was used to compare differences in continuous variables among groups if the data did not conform to a normal distribution. Categorical variables were presented as percentage and compared between groups using Chi-squared test or Fisher’s exact test. Post hoc analysis of postoperative NRS was analyzed using Tukey’s Honest test to identify significant differences between groups. The p-values of < 0.05 were considered statistically significant. This study was conducted utilizing an intention-to-treat analysis approach.

## Results

**Fig. 1:**
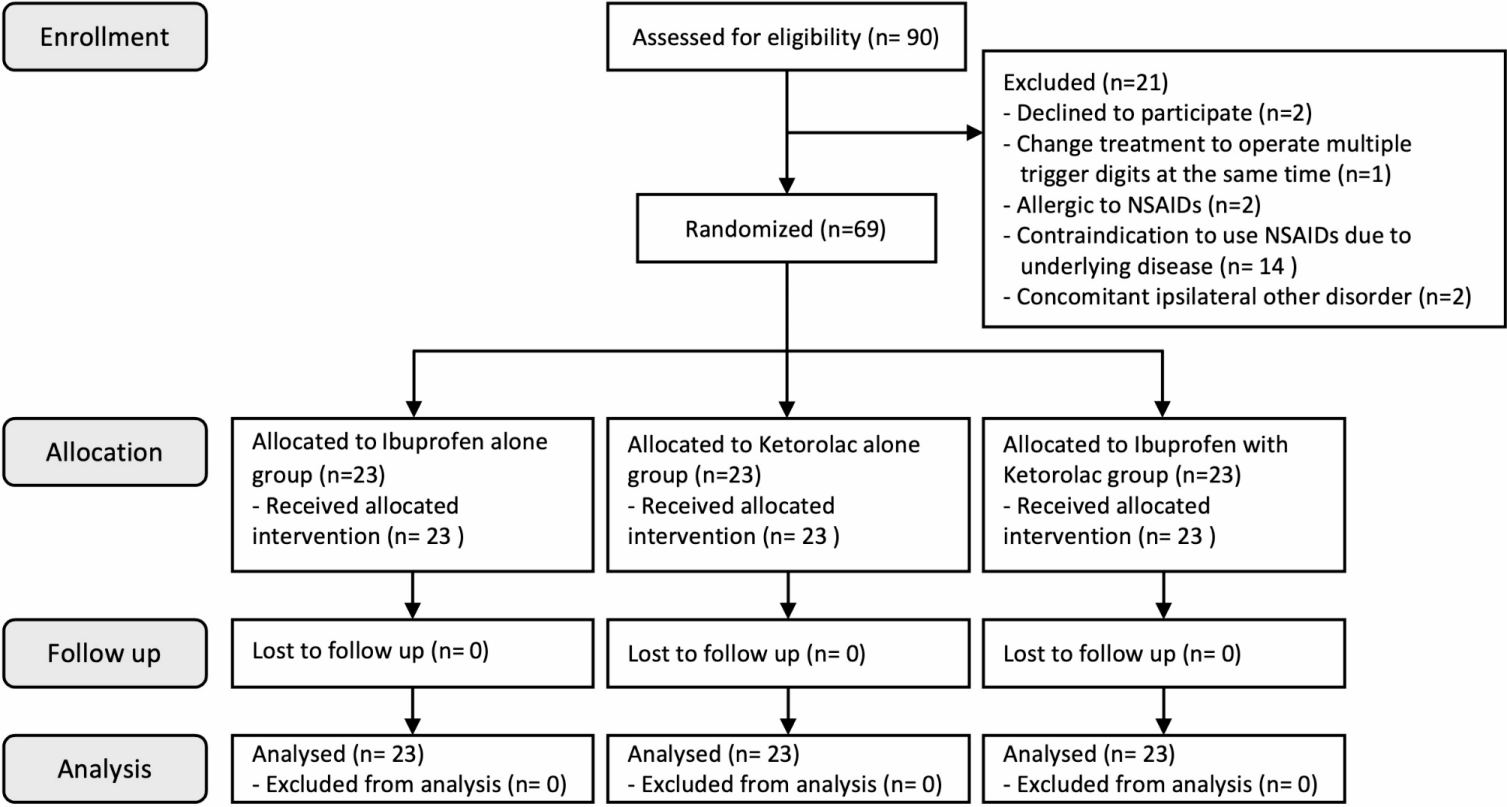
The Consolidated Standards of Reporting Trials (CONSORT) flow diagram

A total of 90 patients with trigger finger were screened for eligibility between December 2021 and October 2022. Among them, 21 patients were excluded from the study based on the predefined exclusion criteria. One patient was planned for single digit trigger finger surgery but later changed the operation to multiple trigger finger surgery. Two patients were allergic to NSAIDs. Two patients had other disorders on the same hand. We excluded 14 patients who had contraindication to use NSAIDs. As a result, a total of 69 eligible patients were enrolled and randomly assigned into three groups. All groups equally consisted of 23 patients. The CONSORT flow diagram of this study was shown in Fig. 1.

The baseline characteristics of participants in each treatment group are summarized in Table 1. Overall, the characteristics were similar between the three groups. Most of the participants were male, constituting the majority in each group. The dominant hand of the participants was predominantly right-handed across all groups. The severity of trigger finger, classified by the Quinell grading system, showed no significant differences between the groups. However, most patients in all groups had Quinell grade 3 trigger finger. Preoperative pain scores at rest and during finger movement were comparable between the groups, with no significant differences observed. The preoperative status of the affected digit, as assessed by ROM of the PIP joint and grip strength, was also similar across the three groups.

**Table 1:** Demographic data between groups

Demographic data	NSS + Ibuprofen (*N* = 23)	Ketorolac + Placebo (*N* = 23)	Ketorolac + Ibuprofen (*N* = 23)	*p*-value
Age* (year)	58.5 (11.6)	54.5 (7.6)	58.2 (8)	0.281^a^
Female	18 (78%)	19 (83%)	17 (74%)	0.774^b^
BMI* (kg/m^2^)	24.8 (4.3)	25.2 (6.5)	24.6 (4.5)	0.926^a^
Involved finger				
- Thumb	8 (35%)	11 (48%)	7 (30.5%)	
- Index finger	2 (8.5%)	2 (8.5%)	2 (8.5%)	
- Middle finger	11 (48%)	8 (35%)	9 (39%)	0.809^b^
- Ring finger	2 (8.5%)	2 (8.5%)	4 (17.5%)	
- Small finger	0	0	1 (4.5%)	
Grading of trigger finger				
- Grade 2	4 (17%)	6 (26%)	8 (35%)	
- Grade 3	10 (44%)	12 (52%)	12 (52%)	0.302^b^
- Grade 4	9 (39%)	5 (22%)	3 (13%)	
Pre-operative NRS pain at rest**	1 (0, 4)	1 (0, 3)	0 (0, 2)	0.502^c^
Pre-operative NRS pain during movement**	7 (5, 7.5)	7 (5, 8)	7 (3, 8.5)	0.838^c^
Pre-operative DASH score**	18.3 (11.7, 25.8)	23.3 (17.9, 30.9)	15 (9.2, 25.4)	0.180^c^
Pre-operative grip strength** (kg)	16 (12, 20.5)	18 (11.5, 20)	16 (9.5, 26)	0.996^c^
Pre-operative ROM of PIP joint** (degree)	77 (62.5, 95)	67 (37.5, 92.5)	85 (65, 92.5)	0.571^c^
Operative time** (min)	15 (10, 20)	20 (10, 20)	20 (10, 25)	0.329^c^

*Data were presented as mean and standard deviation (SD)

**Data were presented as median and interquartile range

^a^ One-way ANOVA test; ^b^ Chi-square test; ^c^ Kruskal-Wallis test

BMI = body mass index; NRS = numerical rating scale; DASH = Disabilities of the arm, shoulder, and hand; ROM = range of motion; PIP = proximal interphalangeal

The postoperative pain evaluation results are presented in Table 2. The NRS pain scores during movement at 6 h after the surgery were significantly different between the groups (p-value = 0.001). At 6 h after surgery, the median NRS pain scores during movement in the Ketorolac alone group and ketorolac with Ibuprofen group were lower compared to the Ibuprofen alone group. When comparing the NRS pain scores during movement at 6 h after surgery between the Ketorolac with Ibuprofen group and the Ketorolac alone group through post hoc analysis, it was found that the Ibuprofen alone group had significantly higher pain score compared to the Ketorolac with Ibuprofen group and Ketorolac alone group (Table 3). At 6 h after surgery, the median pain score at rest of Ibuprofen alone group was higher compared to the other two groups. However, there was no statistically significant difference in pain scores at rest between the groups at this time point. At 24 h, 48 h, 1 week, 2 weeks, 6 weeks and 3 months after the surgery, the differences of median NRS pain scores during movement and at rest were not statistically significant.

**Table 2:** Postoperative outcomes between groups

Postoperative Outcomes	NSS + Ibuprofen (*N* = 23)	Ketorolac + placebo (*N* = 23)	Ketorolac + Ibuprofen (*N* = 23)	*p*-value
NRS pain at rest*				
- 6 - 24 h - 48 h - 1 week - 2 weeks - 6 weeks - 3 months	2 (1, 3) 1 (0, 1.5) 0 (0, 0) 0 (0, 0) 0 (0, 0) 0 (0, 0) 0 (0, 0)	1 (0.5, 2) 1 (0, 1) 0 (0, 1) 0 (0, 0) 0 (0, 0) 0 (0, 0) 0 (0, 0)	1 (0, 3) 2 (0, 2.5) 0 (0, 1) 0 (0, 0) 0 (0, 0) 0 (0, 0) 0 (0, 0)	0.166 0.901 0.164 0.3 0.059 0.352 0.362
NRS pain during finger movement*				
- 6 - 24 h - 48 h - 1 week - 2 weeks - 6 weeks - 3 months	5 (4, 6) 3 (2, 6) 2 (1, 3) 2 (0.5, 3) 2 (1, 3.5) 1 (0, 1.5) 0 (0, 0)	3 (1.5, 3.5) 3 (1.5, 4) 2 (1, 2.5) 1 (0, 2.5) 1 (0.5, 2.5) 1 (0, 1.5) 0 (0, 1)	3 (1.5, 4.5) 3 (2, 4) 3 (1.5, 5) 1 (0, 3) 1 (0.5, 2) 1 (0, 2) 0 (0, 0)	0.001 0.487 0.235 0.926 0.465 0.846 0.128
DASH score*				
- 1 week - 6 weeks - 3 months	11.7 (7.5, 20.2) 3.3 (1.7, 10) 1.3 (0, 1.7)	13.3 (11.7, 17.4) 8.3 (4.2, 10.4) 1.7 (1.3, 3)	13.3 (6.3, 19.4) 5.8 (1.7, 7.8) 1.7 (0, 2.1)	0.674 0.316 0.322
Grip strength* (kg)				
- 1 week - 6 weeks - 3 months	10 (10, 15) 18 (14, 20.5) 21 (19, 24)	13 (9, 14) 16 (13, 20) 20 (18, 24)	12 (6, 20) 18 (14, 22) 22.5 (19, 28)	0.649 0.384 0.216
ROM of PIP joint* (degree)				
- 1 week - 6 weeks - 3 months	75 (57.5, 85) 80 (65, 92.5) 95 (70, 100)	78 (52, 88) 95 (65, 100) 95 (70, 100)	84.5 (70, 95) 97.5 (70, 105) 105 (75, 108)	0.431 0.406 0.272
Complications				
- Ecchymosis - Numbness - GI disturbance - Wound dehiscence	2 (8.7%) 0 2 (8.7%) 0	4 (17.4%) 0 2 (8.7%) 1 (4.3%)	6 (26.1%) 1 (4.3%) 0 0	0.298 0.363 0.346 0.363

*Data were presented as median and interquartile range

NRS = numerical rating scale; DASH = Disabilities of the arm, shoulder, and hand; ROM = range of motion; PIP = proximal interphalangeal; GI = Gastrointestinal

**Table 3:** Post hoc analysis of NRS during finger movement at 6 h postoperative period

Group comparisons	Mean difference	*p*-value^a^	95% Confidence interval	
			Lower bound	Upper bound
NSS + Ibuprofen VS Ketorolac alone	2.3	0.002	0.78	3.83
NSS + Ibuprofen VS Ketorolac + Ibuprofen	1.65	0.031	0.13	3.18
Ketorolac alone VS Ketorolac + Ibuprofen	-0.65	0.564	-2.18	0.87

^a^ Tukey’s Honest Significant Difference test

NRS = numerical rating scale; NSS = normal saline solution

In terms of functional outcomes of the patients, postoperative DASH score, grip strength, and ROM of the PIP joint did not show statistically significant differences between the three treatment groups.

The compliance of postoperative pain medication was assessed by the consumption of Ibuprofen which was similar between the Ketorolac with Ibuprofen group and the Ibuprofen alone group. As for the postoperative consumption of paracetamol as a rescue painkiller, there was no statistically significant difference between the groups.

Regarding postoperative complications, there was no statistically significant difference between the groups. Only one case of numbness over the operated digit was reported in the Ketorolac with Ibuprofen group. There was an increased number of patients who had ecchymosis around surgical site in Ketorolac-receiving groups; however, there was no statistically significant difference between groups. Those complications were eventually resolved within 2 weeks.

## Discussion

This randomized controlled trial is the first to compare local NSAID infiltration with Ketorolac to conventional oral painkillers for patients undergoing open trigger finger release surgery. Previous research on local NSAID infiltration in such surgeries is limited, with most studies focusing on major surgeries. One study showed that local Ketorolac infiltration at the surgical site reduced pain scores, delayed the need for rescue pain medication, and decreased postoperative pain medication use in hand surgery, without significant complications [15]. Thus, local Ketorolac infiltration is both effective and safe for postoperative pain control.

In this study, local infiltration with 30 mg of ketorolac was administered before surgical wound closure. The main finding was a significant reduction in postoperative pain during finger movement at 6 h after administration. Post hoc analysis revealed a statistically significant difference in pain scores between the group receiving only ibuprofen and the groups using local ketorolac infiltration. This suggests that local ketorolac infiltration, whether combined with postoperative NSAIDs or not, effectively reduces pain at the surgical site, especially within the first 6 h post-surgery. This finding suggests that early finger motion can be performed without increasing pain, potentially boosting patients’ confidence and encouraging greater engagement in postoperative rehabilitation. Based on prior research examining the plasma concentration of Ketorolac following local infiltration analgesia, the time to reach peak concentration ranges from 30 min to 4 h. This interval corresponds to the onset of Ketorolac’s therapeutic effects when administered locally [16]. Our results were comparable with previous studies that showed local wound infiltration with ketorolac helped with postoperative pain control [7, 9–11, 14, 15]. 

Open trigger finger release surgery is considered a minor surgery. Postoperative pain might be lower than other major surgeries. Therefore, the pain score between groups might be too small to detect the clinically significant difference. When comparing the duration of pain control after local infiltration of Ketorolac to other research, the duration of pain control from our study was effective only for a shorter period. This might be because we used only 1 ml of 30 mg of ketorolac instead of combining it with local anesthetics such as Bupivacaine and lidocaine, which might prolong the duration of pain relief as found in other literatures. More research about the dose-response relationship and combination of Ketorolac to other drugs could be conducted to prolong the analgesic effect in trigger finger surgery.

Some participants experienced dorsal PIP joint tenderness. According to Monteerarat Y., et al. mentioned that dorsal PIP joint tenderness is more common in trigger fingers and is associated with higher and prolonged postoperative pain after A1 pulley release [17]. To address potential confusion between pain at the surgical site and dorsal PIP pain, the assessors must thoroughly confirm the patients while evaluating the pain specifically over the A1 pulley area or the PIP joint pain.

We also evaluated the functional outcomes of the participants using the DASH score, grip strength, and ROM of the PIP joint. However, the difference was too small to detect statistical significance between groups. Oral NSAIDs and local NSAIDs infiltration showed no statistically significant difference in terms of the functional outcomes.

The complications between the three groups were similar. This result was comparable to previous studies that found no evidence of serious complications [8–11]. We observed 12 participants who experienced ecchymosis around the surgical wound, with 6 participants in the Ketorolac with Ibuprofen group, which had the highest number among the three groups. However, there was no statistical significance between groups. All ecchymosis around surgical wounds were spontaneously resolved within 2 weeks without disturbing their functions. In this study, we only assessed gastrointestinal disturbance symptoms as potential systemic complications and found no statistically significant differences between groups. However, we did not collect blood samples from the patients to identify any other potential systemic complications, which might be a limitation of this study.

## Conclusion

Local infiltration of 30 mg of ketorolac at the surgical site demonstrated superior pain control during finger movement within 6 h after trigger finger release surgery compared to oral NSAIDs. This method may be considered an alternative pain control option for patients who have contraindications to systemic NSAIDs.

## Data Availability

The datasets used or analyzed during the study are available from the corresponding author on reasonable request.
